# 1st International Symposium on Women in Tunicate Biology: Meeting report

**DOI:** 10.1002/dvg.23571

**Published:** 2023-11-20

**Authors:** Anna Di Gregorio, Marie L. Nydam

**Affiliations:** 1 Department of Molecular Pathobiology, New York University College of Dentistry, New York, New York, USA; 2 Life Sciences Concentration, Soka University of America, Aliso Viejo, California, USA

**Keywords:** ascidian, meeting report, tunicate, women scientist

The 1st International Symposium on Women in Tunicate Biology was held online on March 28 and 29, 2023. This global symposium was attended by 45–50 researchers from countries including Austria, Brazil, India, Italy, Japan, New Zealand, Turkey, and the United States. Figure 1 is a photograph of some of the participants from the March 28th session.

The main goals of this symposium were honoring women who advanced the field of tunicate biology, sharing current research interests, promoting collaborations, inspiring and supporting new and aspiring independent investigators, and fostering inclusivity. The symposium started its first day with biographical presentations of women scientists who pioneered the field, followed by tributes to recently retired female ascidiologists. The second day was mainly dedicated to presentations on the research currently being conducted by female principal investigators. On the first day, Anna Di Gregorio gave an introduction on the general history of tunicate research and then highlighted women who were active in the 19th and 20th centuries: Gladys Amelia Anslow from the United States, an accomplished physicist and first woman to work with the cyclotron at the University of California at Berkeley, who studied the effect of copper ions on ascidian metamorphosis; Helga Henriette Lindel Zwillenberg from Germany, who obtained the first images of chromosomes in ascidians through a method that she perfected for these organisms; Nel Krijgsman, from the Netherlands, who studied the pacemakers of the *Ciona* heart and the effects of different neurotransmitters on their function, and Winifred Parsons, who studied the transport of carbon dioxide in the blood of ascidians and other vertebrates during her residence at the Stazione Zoologica in Napoli (Naples), Italy.

There were three talks honoring Italian women researchers. Fiorenza De Bernardi presented a tribute to Giuseppina Ortolani, who gained international recognition for her lineage-tracing experiments in solitary ascidians and trained numerous students who later became leaders in various fields of tunicate biology. Lucia Manni presented on her mentor Giovanna Zaniolo, another exceptionally talented experimentalist who pioneered studies of regeneration, allorecognition, and aging in colonial ascidians. Filomena Ristoratore and Annamaria Locascio described the research interests and accomplishments of recently retired women scientists from the Stazione Zoologica in Napoli: Margherita Branno, Anna Palumbo, Rosaria De Santis, and Elisabetta Tosti. Then, Megan Wilson introduced Beryl Brewin, a taxonomist and ecologist from New Zealand who worked for nearly 30 years at the University of Otago. Brewin described more than 80 ascidian genera and species from Australia and New Zealand and made a large financial donation in her will to support the Portobello Marine Laboratory. Megan also presented her lab’s research, discussing in particular the insights her group has gained from genomics, transcriptomics, and DNA accessibility mapping in understanding the phenomenon of whole-body regeneration in *Botrylloides leachii*. At the end of her talk, Megan described the challenges encountered by her lab, and the entire research community in New Zealand, during the lengthy lockdowns that were enforced due to the COVID-19 pandemic.

Next, Jhimli Mondal introduced the life and work of V. K. Meenakshi, an ascidian taxonomist from India who inspired a generation of biologists. Prof Meenakshi was the first Indian woman to work on species of ascidians found on Indian coasts. She and her collaborators published new records for several hundred species found in India, and described 10 new species, some of which are featured in Figure 2. Dr Meenakshi joined the symposium online and several of her students attended the symposium to honor her mentorship, share their appreciation of this initiative, and participate in the other presentations.

Finally, Marie Nydam highlighted the research and educational efforts of Gretchen Lambert from the United States. Together with her beloved husband Charles, Lambert has been publishing on many aspects of ascidian biology, ecology, and taxonomy since 1968. The extraordinary research efforts of Gretchen and Charles Lambert have been honored by other ascidiologists, who have named newly discovered species after them. Some examples are shown in Figure 3, which also includes a new species, *Trididemnum alexi*, lovingly named by Gretchen in honor of her grandson, Alex Coleman.

Gretchen has also been an enthusiastic mentor and educator, organizing and teaching at least 25 taxonomy workshops since 2001. Twice a year (for nearly 50 years!), Gretchen has been preparing “Ascidian News,” a bulletin that, as she politely clarifies in each issue, “is not part of the scientific literature,” but contains a helpful list of recently published manuscripts and provides an unofficial platform to anyone who wants to share ongoing projects, announcements, publications, and accomplishments with the tunicate scientific community at large.

On the second day of the symposium, female principal investigators gave summaries of the ongoing research in their laboratories. Many of these PIs introduced the female postdocs, students, and staff who had conducted or supported the work in their laboratories. Izumi Oda-Ishii summarized the work of female ascidian researchers in Japan. She described the research of Kaoru Imai and Miki Tokuoka on gene regulatory networks in *Ciona* embryogenesis, the ongoing work of Atsuko Sato on *Ciona robusta* and *Ciona intestinalis* and the hybrids that these related species can generate, and the research of Kogiku Shiba on the biochemical and biophysical bases of sperm flagellar motility. Of note, all of these researchers provided individual contributions to this special issue, which describe in detail their fascinating research. Oda-Ishii then discussed her own work on *Zic-r.a*, a gene that encodes a transcription factor that is essential for the development of muscle cells in *C. robusta* embryos and is also expressed in the nervous system, and described her findings on the molecular mechanisms through which this transcription factor is able to control gene expression in these different tissues.

Following this presentation, Arzu Karahan explained the wide variety of *Botrylloides* research projects in her laboratory at the Institute of Marine Sciences, Middle East Technical University, in Erdemli, Turkey. Her lab’s research includes species identification, life cycle monitoring, ecogenomics, whole-genome and transcriptome sequencing, genetic interactions during whole-body regeneration, and stem-cell aging. Susanna Lopez-Legentil, from the United States, focused on her recent work at the University of North Carolina, Wilmington: creating an inventory of local ascidian species, examining their relative abundance before and after a hurricane disturbance, cataloging ascidian diversity and abundances in seagrass beds, and comparing microbial symbionts between ascidian populations. Next, Ayelet Voskoboynik from Stanford University (USA) honored the work of Virginia (Ginny) Scofield, Kathi Ishizuka, and Karla Palmeri on allorecognition in *Botryllus schlosseri*, and gave an overview of the far-reaching research that she is leading on stem cell niches, stem cell aging, central nervous system development and degeneration, changes in gene expression of circadian clock genes in aging animals. She also described the new method for high-throughput sequencing that she and her collaborators developed and used to sequence and assemble *the B. schlosseri* genome.

Finally, Billie Swalla from the University of Washington (USA) took the audience on her career journey, from her fascination with ascidian embryos as an undergraduate to becoming the first female director of Friday Harbor Laboratories. She highlighted her laboratory’s work on *Boltenia villosa*, *Corella inflata*, and *Molgula* spp. development, distribution of native and non-native species in the Salish Sea, honored her mentor Mary Rice (Director of the Smithsonian Marine Station in Fort Pierce, Florida, USA, from 1981 to 2002) and offered valuable advice for young female scientists.

Anna Di Gregorio concluded the symposium with a few remarks on the goals of this initiative. She advocated that the community preserve and advertise the work of women who advanced the field of tunicate biology in our publications and websites, and shared her hope that these presentations will promote collaborations among existing labs and inspire younger researchers to carry on the legacy of their research on tunicate biology. For the goal of fostering inclusivity, it was suggested that members of the tunicate community increase their interactions, for example, by co-writing papers, review articles and grant applications, and jointly reach out to members of underrepresented groups. The symposium ended with a discussion about the structure and timing of future symposia led by women tunicate researchers; the online format was overwhelmingly appreciated, as it allowed interactions without any financial burden. There was also consensus that these symposia should continue on alternating years with the International Tunicate Meetings, and that future iterations of this gathering could be more focused on the research carried out by postdocs and graduate students and devote one of the time slots available to the discussion of work-life balance for all scientists with child-rearing and domestic responsibilities.

The proceedings of the symposium are grouped in this Special Issue of genesis, *The Journal of Genetics and Development*. The manuscripts in this collection include “tribute” papers that honor women scientists who pioneered and advanced the field of tunicate biology, as well as “In Her Words” letters, which provided a canvas for women scientists to freely illustrate their research, and themselves. We are profoundly grateful to Prof Jean-Pierre Saint-Jeannet, our Editor-in-Chief, for providing us with the unprecedented opportunity to compile this issue and for his unwavering support, to the colleagues who participated in this initiative, and to those who cheered from the sidelines.

Despite our best efforts, neither the symposium nor this collection could possibly include all former, current, and budding female ascidiologists. We humbly suggest that our initiative might be the first of many related celebrations of the work and achievements of female scientists in tunicate biology and inspire related celebrations of women scientists in other fields of research.

## Figures and Tables

**FIGURE 1:**
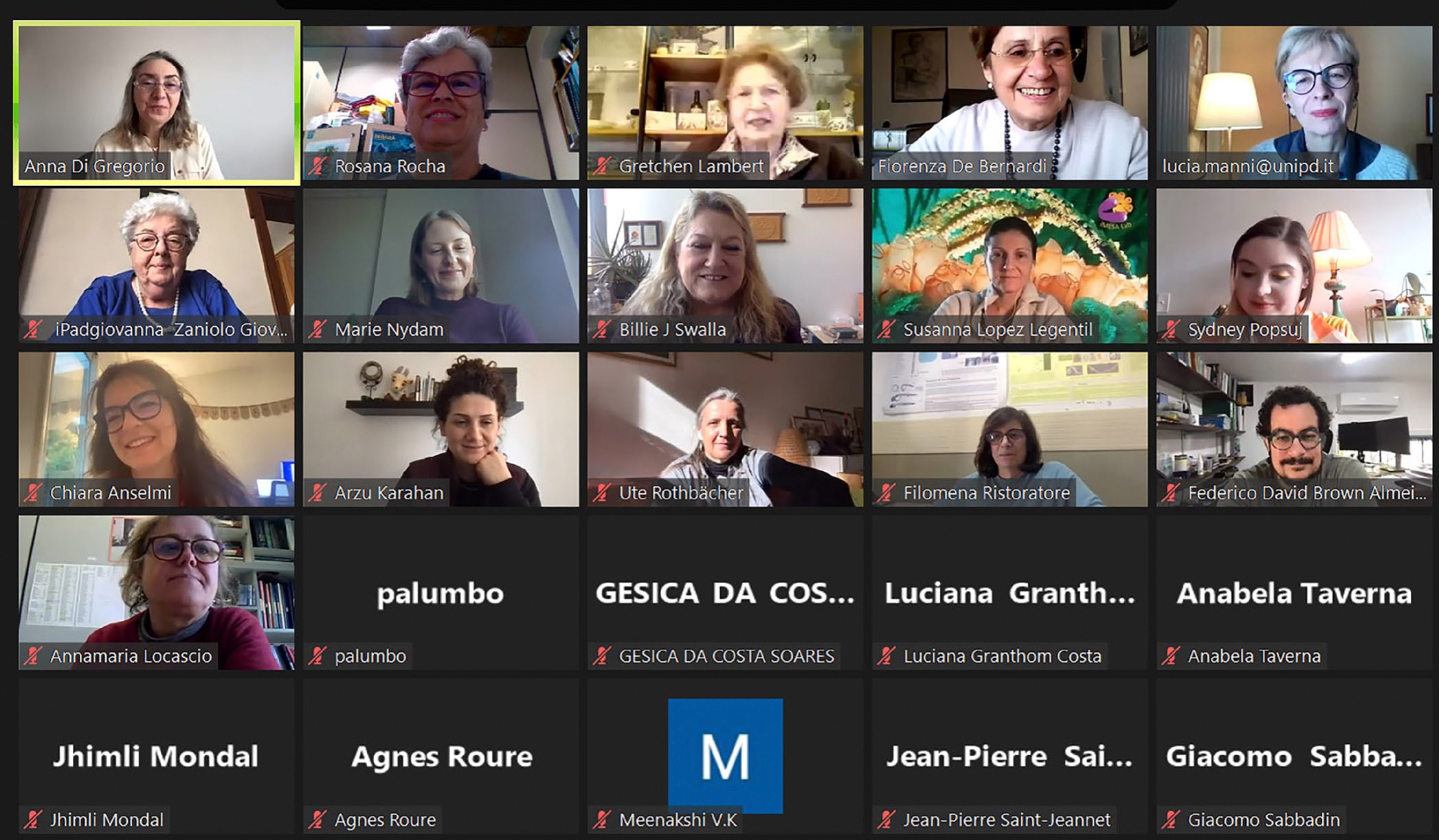
A worldwide community gathered online to celebrate the contributions of women to the field of tunicate biology. Screenshot courtesy of Prof Rosana Rocha.

**FIGURE 2:**
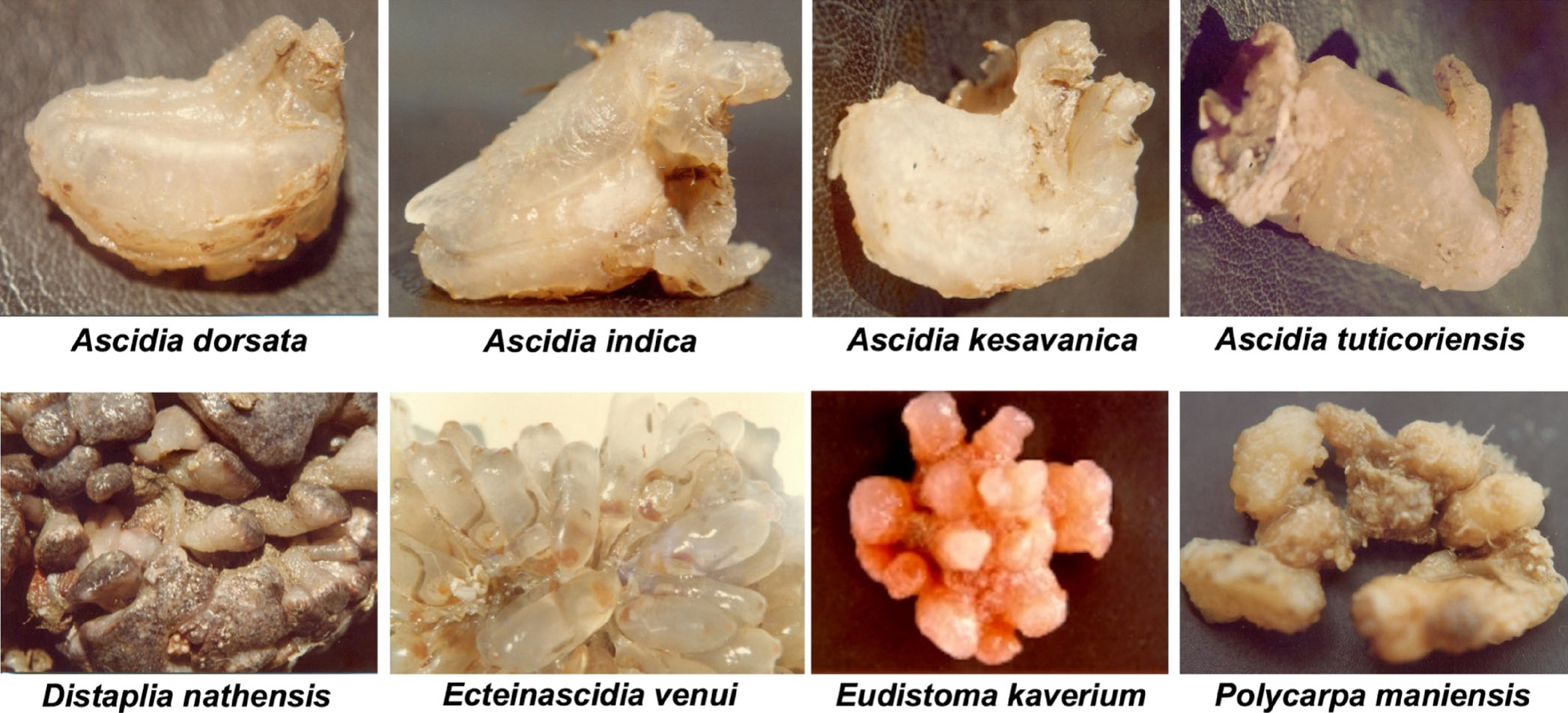
A few examples of new ascidian species described by Prof V. K. Meenakshi. Reproduced with permission from Meenakshi and Senthamarai (2013), modified using original photographs kindly provided by Prof V. K. Meenakshi and Dr Jhimli Mondal.

**FIGURE 3:**
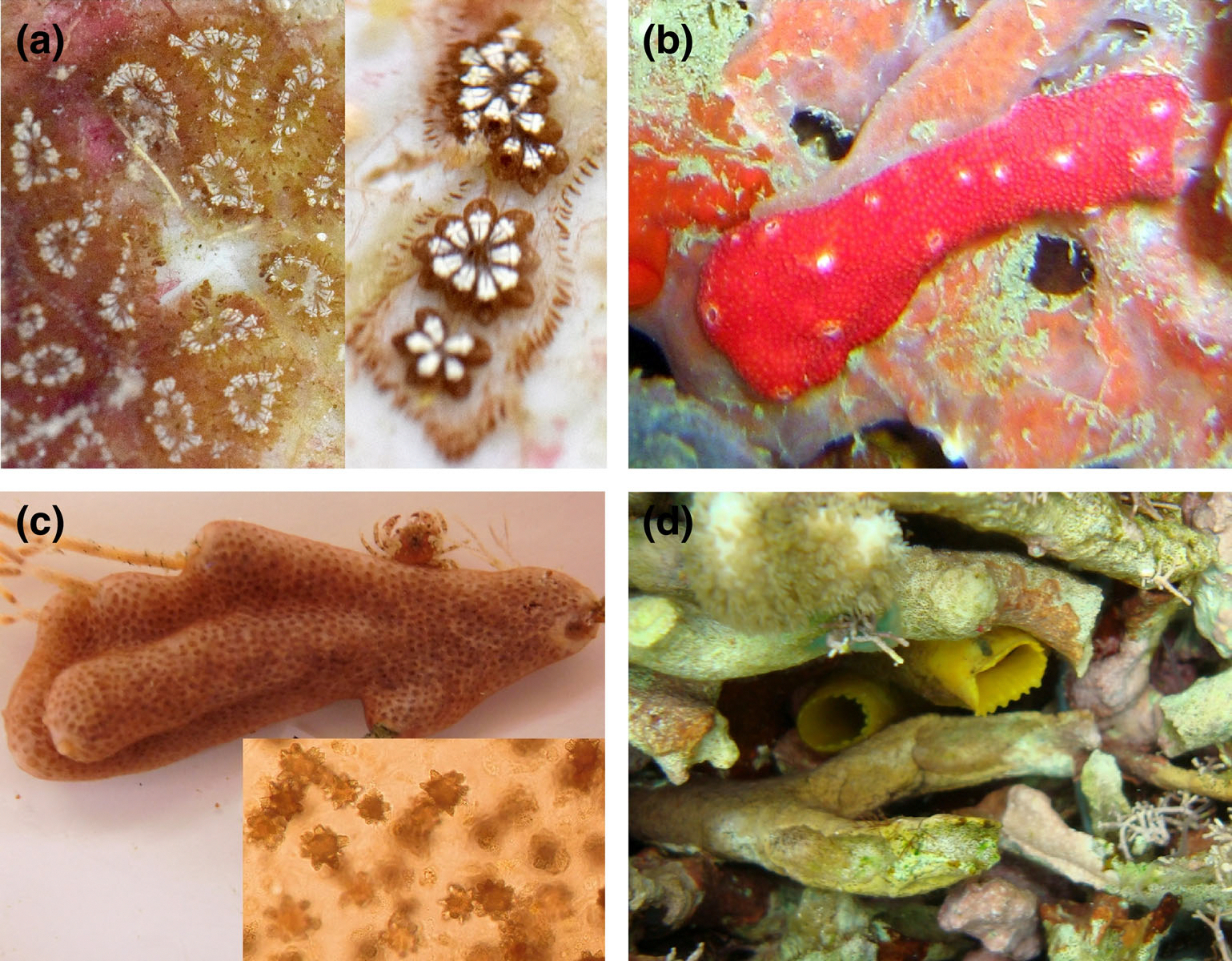
Ascidian species named after ascidiologists…and their loved ones. (a) *Botryllus lambertorum*, discovered and described by Marie Nydam, Lilian Palomino-Alvarez, and Rosana Rocha, and dedicated to Gretchen and Charles Lambert (Palomino-Alvarez et al., 2022). (b) *Didemnum lambertae*, discovered by Rosana Rocha (Rocha et al., 2015). (c) *Trididemnum alexi*, named by Gretchen Lambert in honor of her grandson, Alex (Lambert, 2003). Inset: microphotograph showing a group of calcium carbonate spicules found on the tunic of *T. alexi*. (d) *Ascidia monnioti*, discovered by Rosana Rocha and named after the prominent ascidian taxonomist Françoise Monniot (Bonnet & Rocha, 2011). Note that only the siphons are visible (center), as this species is always hidden in its native habitat. Images in (b, d), kindly provided by Rosana Rocha. Images in (c), courtesy of Gretchen Lambert.
